# Left Ventricular Diastolic Dysfunction in Type 2 Diabetes Mellitus: A Single-Centre Observational Study From a Tertiary Care Hospital in South India

**DOI:** 10.7759/cureus.34667

**Published:** 2023-02-06

**Authors:** Wazeem CM, Gopalakrishna Pillai, Arun Divakar, Renjitha Bhaskaran

**Affiliations:** 1 Internal Medicine, Amrita Institute of Medical Sciences and Research Center, Kochi, IND; 2 Biostatistics, Amrita Institute of Medical Sciences and Research Center, Kochi, IND

**Keywords:** hba1c, south india, prevalence, left ventricular diastolic dysfunction, diabetes mellitus

## Abstract

Background

There is a high prevalence of left ventricular diastolic dysfunction (LVDD) in patients with type 2 diabetes mellitus (T2DM). The influencing factors of LVDD in T2DM are not fully understood.

Objective

This study aimed at assessing the prevalence of LVDD in T2DM as well as looking at the association between various parameters related to T2DM with LVDD in patients with T2DM.

Materials and methods

This was a single-centre cross-sectional study in Kerala, India. The primary objective of the study was to assess the prevalence of LVDD in T2DM. The secondary objectives were to look for an association between higher glycated haemoglobin (HbA1c), complications of T2DM, age, and gender of the patient with the presence of LVDD.

Results

A total of 80 patients were included in the study. There were 40 patients with LVDD with a prevalence of 50%. There was a statistically significant positive association between increased age, longer duration of diabetes, higher HbA1C, the presence of diabetic neuropathy, diabetic retinopathy, and diabetic nephropathy with the prevalence of LVDD. A logistic regression analysis demonstrated that the presence of diabetic retinopathy is a risk factor for LVDD in the study subjects.

## Introduction

In 2021, 536.6 million people aged between 20 to 79 years were estimated to have diabetes with a prevalence of 10.5% with a projected increase to 783.2 million people in 2045 with a prevalence of 12.2% [1]. In the state of Kerala in India, which is thought to have a high prevalence of diabetes, a 10-year prospective cohort study showed an incidence rate of 24.5 per 1000 person-years for type 2 diabetes mellitus (T2DM) and 45.01 per 1000 person-years for impaired fasting glucose [2].

Diabetes can cause structural and functional changes within the heart, even without the development of atherosclerotic disease. Left ventricular diastolic dysfunction (LVDD) is thought to represent one of the first pre-clinical manifestations associated with diabetic cardiomyopathy and can precede systolic dysfunction with the ability to evolve into symptomatic cardiac failure [3]. The underlying mechanism of LVDD is attributed to the development of left ventricular hypertrophy with associated myocardial interstitial fibrosis with subsequent myocardial stiffness as well as diastolic relaxation impairment [4]. Over the course of time, diastolic dysfunction may progress to diastolic heart failure in the absence of intervention and this makes it an important entity to be picked up early.

With a high prevalence of T2DM in our general population, data on the prevalence of LVDD in this subset of the population with T2DM is lacking from South India. Hence, we conducted a single-centre prospective observational study to look at the prevalence of LVDD in T2DM as well as look at the association between various parameters related to T2DM with LVDD in patients with T2DM in Kerala.

## Materials and methods

This was a single-centre cross-sectional study conducted at Amrita Institute of Medical Sciences, Kerala, India for a period of two years between April 2020 and April 2022. The primary objective of the study was to assess the prevalence of LVDD in T2DM. The secondary objectives were to look for an association between higher glycated haemoglobin (HbA1c), complications of T2DM, age, and gender of the patient with the presence of LVDD. Ethical committee clearance was obtained from the institutional review board at Amrita Institute of Medical Sciences and Research Centre (approval no: ECASM-AIMS-2022-254). 

Inclusion and exclusion criteria

A total of 80 patients were included in the study. All patients who fit the inclusion criteria during the pre-determined study period were enrolled in the study after written informed consent. The patients were recruited from the Internal Medicine and Endocrinology department outpatient clinics. The inclusion criteria were patients aged above 30 years with T2DM. Patients were excluded if they had established coronary heart disease, congenital heart disease, valvular heart disease, cardiomyopathy, or any other pre-existing heart conditions. They were also excluded if they were hypertensive, had a poor window on trans-thoracic echocardiography if the patient had Type 1 diabetes or underlying thyroid conditions, or if they were alcoholics, defined as the intake of more than 14 units of alcohol per week.

All patients enrolled in the study had a detailed clinical history, physical examination, and biochemistry tests including a complete blood count (CBC), renal function tests, liver function tests, fasting blood sugar (FBS), post-prandial blood sugar (PPBS), HbA1C, a fasting lipid profile, and a urine protein estimation. Subsequently, they underwent a 2-D directed M mode trans-thoracic echocardiogram (TTE) with a 3.5 MHz transducer using a Philips HD 15 ECHO machine. Inter-performer variability was minimised by having all the TTEs performed by the same two TTE technicians followed by an image review by the same two cardiologists. 

Definitions

T2DM was defined as HbA1c ≥ 6.5 % or random blood sugar (RBS) ≥ 200 mg/dl or FBS ≥ 126  mg/dl or oral glucose tolerance testing (OGTT) showing an OGTT 2‑hour glucose in venous plasma ≥ 200 mg/dl [5]. 

TTE evaluation of LVDD was performed by the measurement of trans-mitral flow parameters which included early (E) and late (A) diastolic filling velocities, and using the E/A ratio. An E/A ratio of 0.75-1.5 was considered normal and any patient with an E/A ratio of less than 0.75 was considered to have LVDD [6]. 

Diabetic nephropathy was defined as the presence of overt macroalbuminuria at a level ≥300 mg/24 hours [7]. Diabetic retinopathy and diabetic neuropathy were clinical diagnoses. 

Statistical analysis

Statistical analysis was performed using IBM SPSS Version 20.0 (IBM Corp., Armonk, NY). Categorical variables were expressed as percentages and frequencies. Continuous variables were expressed as mean and standard deviation. The statistical association was checked using T-tests for continuous variables and Fischer's exact test for categorical variables. A backward elimination stepwise multivariate logistic regression analysis was also performed to study the impact of the studied parameters against the presence of LVDD. A p-value < 0.05 was considered significant.

## Results

A total of 80 patients were enrolled in the study. There were 40 (50%) patients with LVDD while 40 (50%) patients did not have any evidence of LVDD. The mean (M) ± standard deviation (SD) age of the patients was 59.4 ± 9 years. The M ± SD age in the group with LVDD was 59.5 ± 9.9 years. The M ± SD age in the group without LVDD was 55.4 ± 7.5 years. There was a statistically significant association between increased age and the presence of LVDD (p= 0.044). There were 50 (62.5%) males and 30 (37.5%) females in the study. In the group of patients with LVDD, 26 (65%) were males while 14 (35%) were females. In the group without LVDD, 24 (60%) were males while 16 (40%) were females. There was no statistically significant association between gender and the presence of LVDD (p=0.82). The M ± SD duration of diabetes in the study was 13.3 ± 6.7 years. The M ± SD duration of diabetes in the group with LVDD was 15 ± 6.3 years. The M ± SD duration of diabetes in the group without LVDD was 11.7 ± 6.8 years. There was a statistically significant association between increased duration of diabetes and the presence of LVDD (p= 0.015)

With respect to evidence of diabetic neuropathy, 31 (38.8%) patients had diabetic neuropathy while 39 (61.2%) patients did not. In the group of patients with LVDD, 23 (57.5%) had diabetic neuropathy while 17 (42.5%) did not. In the group without LVDD, 8 (20%) had diabetic neuropathy while 32 (80%) did not. There was a statistically significant association between diabetic neuropathy and the presence of LVDD (p=0.001). With respect to evidence of diabetic retinopathy, 20 (25%) patients had evidence of retinopathy while 60 (75%) patients did not. In the group of patients with LVDD, 17 (42.5%) had diabetic retinopathy while 23 (57.5%) did not. In the group without LVDD, 3 (7.5%) had diabetic retinopathy while 37 (92.5%) did not. There was a statistically significant association between diabetic neuropathy and the presence of LVDD (p<0.001). With respect to evidence of diabetic nephropathy, 17 (21.3%) patients had evidence of diabetic nephropathy while 63 (78.7%) patients did not. In the group of patients with LVDD, 14 (35%) had diabetic nephropathy while 26 (65%) did not. In the group without LVDD, 3 (7.5%) had diabetic nephropathy while 37 (92.5%) did not. There was a statistically significant association between diabetic nephropathy and the presence of LVDD (p=0.005). 

The M ± SD HbA1C was 8.8 ±1.8 %. The M ± SD HbA1C in the group with LVDD was 9.5 ± 1.9 %. The M ± SD HbA1C in the group without LVDD was 8.2 ± 1.4 %. There was a statistically significant association between increased HbA1C and the presence of LVDD (p= 0.003). The M ± SD FBS in the study was 173.2 ± 69.9 mg/dL. The M ± SD FBS in the group with LVDD was 183.5 ± 80.1 mg/dL. The M ± SD FBS in the group without LVDD was 162.8 ± 57.2 mg/dL. There was no statistically significant association between FBS and the presence of LVDD (p= 0.275). The M ± SD PPBS in the study was 282.1 ± 88 mg/dL. The M ± SD PPBS in the group with LVDD was 295.3 ± 93.6 mg/dL. The M ± SD PPBS in the group without LVDD was 269 ± 81.2 mg/dL. There was no statistically significant association between PPBS and the presence of LVDD (p= 0.184). Various study parameters and associations are summarised in Table 1.

**Table 1:** Table showing the various study parameters ^1^ Expressed as mean ± standard deviation LVDD: left ventricular diastolic dysfunction; HbA1c: Glycated haemoglobin; FBS: Fasting blood sugar; PPBS: Post-prandial blood sugar

Parameter	LVDD group	No LVDD group	p-value
Age ^1^(years)	59.5 ± 9.9	55.4 ± 7.5	0.044
Male gender	26 (65%)	24 (60%)	0.82
Female gender	14 (35%)	16 (40%)	
Duration of diabetes ^1 ^(years)	15 ± 6.3	11.7 ± 6.8	0.015
Diabetic neuropathy	23 (57.5%)	8 (20%)	0.001
Diabetic retinopathy	17 (42.5%)	3 (7.5%)	<0.001
Diabetic nephropathy	14 (35%)	3 (7.5%)	0.005
HbA1C ^1^(percentage)	9.5 ± 1.9	8.2 ± 1.4	0.003
FBS ^1^ (mg/dL)	183.5 ± 80.1	162.8 ± 57.2	0.275
PPBS ^1^ (mg/dL)	295.3 ± 93.6	269 ± 81.2	0.184

A backward elimination stepwise multivariate logistic regression analysis showed a significant association between the presence of diabetic retinopathy with the presence of LVDD (p=0.001). No other studied parameters were found to have a significant association with the presence of LVDD. The stepwise backward elimination regression analysis is summarised in Table 2.

**Table 2:** Backward elimination stepwise multivariate logistic regression analysis T2DM: Type 2 diabetes mellitus; HbA1c: Glycated haemoglobin

Backward elimination steps		Logistic regression coefficient	Standard error	significance (p-value)	Odds ratio	95% Confidence Interval	
						Lower	Upper
Step 1	Age	.027	.033	.418	1.027	.962	1.097
	Duration of T2DM	.017	.042	.689	1.017	.936	1.105
	HbA1C	.652	.606	.282	1.919	.585	6.291
	Diabetic Neuropathy	.537	.653	.411	1.711	.476	6.147
	Diabetic Retinopathy	1.253	.822	.127	3.502	.699	17.538
	Diabetic Nephropathy	.782	.854	.359	2.186	.410	11.649
	Constant	-2.854	1.881	.129	.058		
Step 2	Age	.031	.032	.324	1.032	.970	1.098
	HbA1C	.654	.605	.279	1.924	.588	6.299
	Diabetic Neuropathy	.533	.652	.414	1.705	.475	6.123
	Diabetic Retinopathy	1.289	.816	.114	3.627	.733	17.962
	Diabetic Nephropathy	.849	.834	.308	2.338	.456	11.979
	Constant	-2.897	1.883	.124	.055		
Step 3	Age	.035	.031	.255	1.036	.975	1.101
	HbA1C	.773	.589	.189	2.167	.683	6.872
	Diabetic Retinopathy	1.506	.762	.048	4.507	1.012	20.067
	Diabetic Nephropathy	1.041	.794	.190	2.833	.598	13.427
	Constant	-3.107	1.852	.093	.045		
Step 4	HbA1C	.751	.587	.201	2.119	.670	6.697
	Diabetic Retinopathy	1.709	.743	.021	5.523	1.288	23.680
	Diabetic Nephropathy	.970	.785	.217	2.638	.566	12.297
	Constant	-1.102	.514	.032	.332		
Step 5	HbA1C	.844	.588	.151	2.326	.734	7.372
	Diabetic Retinopathy	2.086	.688	.002	8.049	2.092	30.972
	Constant	-1.087	.519	.036	.337		
Step 6	Diabetic Retinopathy	2.210	.680	.001	9.116	2.403	34.577
	Constant	-.475	.266	.073	.622		

## Discussion

This was a single-centre observational study aimed at studying the association between T2DM and LVDD in patients with T2DM in Kerala, India. Our study had an equal split of patients with T2DM who did and did not have LVDD which suggests that one in two patients with T2DM have LVDD, a prevalence of 50%. Yadava et al. found a very similar prevalence for LVDD of 47.8% in a cohort of patients with T2DM in a study from Nepal [8]. In a large systematic review, Bouthoorn et al. also found a similar prevalence for LVDD where it was 48% for hospital patients with T2DM and 35% for patients with T2DM in the community [9]. This suggests that all patients with T2DM should undergo screening for LVDD. 

We found that increasing age, longer duration of T2DM, the presence of diabetic neuropathy, diabetic retinopathy, diabetic nephropathy, and higher HbA1C values correlated with the presence of LVDD. Yadava et al. also found an association between LVDD with increasing age and longer duration of T2DM while they failed to demonstrate an association with higher HbA1C [8]. Kurioka et al. from Japan found significant associations between increasing age, increasing diabetes duration, and diabetic retinopathy with the prevalence of LVDD [10].

However, in our study, on a multivariate logistic regression analysis, only the presence of diabetic retinopathy was found to be associated with LVDD. Interestingly, in Kurioka et al.'s study as well a regression analysis demonstrated a significant correlation only between increasing age and increasing grades of diabetic retinopathy with the presence of asymptomatic diastolic dysfunction [10]. These findings suggest that in the setting of a microvascular complication of T2DM such as retinopathy, the importance of assessing for underlying LVDD increases and there should be an active effort to look for LVDD in these patients. Establishing the presence of retinopathy is also a quick and economical process that can be done in a clinical setting and this could provide a clue toward associated cardiovascular complications.

In contrast, however, Poulsen et al. [11] found a prevalence of 40% for LVDD in T2DM and in addition found a significantassociation between the presence of LVDD and abnormal myocardial perfusion. They however found a much weaker association between LVDD and vascular function. Based on this, they suggested that LVDD in T2DM was associated with intrinsic LV dysfunction rather than due to vascular disease or arterial stiffening. These findings, coupled with the association we found in our study to diabetic retinopathy, could indicate that LVDD in T2DM could be multi-factorial with both intrinsic LV dysfunction along with arterial or vascular dysfunction playing a combined role.

Our study had several limitations. Although the study was designed before the COVID-19 pandemic, the study period encompassed the bulk of the pandemic and lockdowns, which resulted in a relatively small sample size. There could also be confounders posed by COVID-19 in the selection of the patients with patients who were actively shielding not having visited the hospital. The diagnosis of LVDD is also done using echocardiography which has an element of inter-performer variability although, in an attempt to minimise this, the same two echo technicians performed all the scans and the same cardiology doctors reviewed the images for all patients. Due to the COVID-19 pandemic and due to the nature of the way the clinics from which the patients were recruited were run during the pandemic with minimal contact, data on the body mass index of the patients could not be captured which could have influenced our results. Finally, this was a cross-sectional study looking at an association between LVDD and various parameters but a longitudinal study would have been preferable to look at these associations.

## Conclusions

There is a high prevalence of left ventricular diastolic dysfunction in T2DM. The presence of LVDD correlated with increasing age, poorer glycemic control, a longer duration of T2DM, and the presence of microvascular complications such as diabetic retinopathy.

Patients with T2DM even without known cardiovascular disease or systemic hypertension should undergo active screening for LVDD, especially in the setting of established retinopathy and advanced age. Further longitudinal studies are necessary to establish the relationship between LVDD and T2DM and associated complications.
